# Singular Phenomena Accompanying Menstruation and Uterine Hemorrhage

**Published:** 1842-01

**Authors:** J. E. Sands

**Affiliations:** Wilson county, Tennessee


Art. VIII.—*Singular Phenomena accompanying Menstruation and Uterine Hemorrhage.* By Dr. J. E. Sands, of Wilson county, Tennessee.

Mrs. B., a native of the State of Georgia, is 56 years old, and has 8 children. She is quite fleshy; has dark eyes, red hair, and a thin, tender, white skin; is of a very lively, cheerful disposition; labors hard and takes a great deal of exercise.

On her left side, commencing about the second false rib, is a line three-fourths of an inch wide which passes directly upwards a little external to the mamma, and stops two inches from the clavicle. Here it divides into three branches of about half its own width; the outer branch, three inches long, passes obliquely upwards and outwards nearly to the scapulo—humeral articulation, where it terminates in a prominence about the size of a half-ounce bullet; the inner one, of the same length, runs obliquely upwards and inwards almost to the junction of the clavicle and sternum, and terminates like the first; the third branch, an inch and a half long, pursues an intermediate course and terminates like the others.

She began to menstruate at the age of 12, and continued to do so regularly until she was 25, when she married; three months afterwards conception took place, and the catamenial discharge ceased. At the usual period she gave birth to a son; two weeks afterwards the menses made their appearance again, and recurred at regular intervals until she was six months advanced in her next pregnancy. The same thing attended all her subsequent pregnancies. At the age of 49 the menses ceased entirely, after which she had no flow of any kind from the uterus until last December, when she suffered with a violent hemorrhage from that organ.

At every menstrual period, from the time menstruation began until it ceased, a fluid resembling the catamenial discharge in all its sensible qualities flowed from the mark or lines described above; the discharge from this part observed precisely all the changes of that from the uterus.

From the birth of her last child, which was in her 38th year, till the final cessation of the menses, she was subject to uterine hemorrhage, and the flow from the side was in direct proportion to that from the uterus, being profuse when the latter was profuse, and ceasing when it ceased. A similar discharge accompanied her last attack of hemorrhage in December.

The skin on these marks or lines is slightly elevated, of a dark red color, assuming somewhat the appearance of an aneurismal tumor. The discharge, to use her own expression, "comes out like the perspiration through the skin." No perspiration is ever discovered on these marks, although she generally perspires freely.

May, 1841.

